# Hemoglobin-albumin-lymphocyte-platelet score associated with diabetic foot severity, unlike modified systemic immune-inflammatory index and modified systemic inflammatory response index

**DOI:** 10.3389/fendo.2026.1823691

**Published:** 2026-05-25

**Authors:** Weiyu Pan, Zhaoyuan Nie, Qizhi Tang, Ling Zhao

**Affiliations:** 1 Department of Endocrinology, Guangdong Provincial Hospital of Integrated Traditional Chinese and Western Medicine Affiliated to Guangzhou University of Chinese Medicine, Guangdong, China; 2 Department of the Traditional Chinese Medicine, The Third Affiliated Hospital of Guangzhou Medical University, Guangdong, China; 3 Department of Endocrinology, The Second Affiliated Hospital of Guangzhou University of Chinese Medicine, Guangdong, China

**Keywords:** diabetic foot, hemoglobin-albumin-lymphocyte-platelet score, modified systemic immune-inflammatory index, modified systemic inflammatory response index, severity

## Abstract

**Background:**

Accurate evaluation of diabetic foot severity and prompt intervention is expected to improve patient prognosis. Systemic inflammatory response and nutritional metabolic status play important roles in the progression of diabetic foot. The purpose of this study was to investigate the relationship between the hemoglobin-albumin-lymphocyte-platelet (HALP) score, modified systemic immune-inflammatory index (mSII), modified systemic inflammatory response index (mSIRI) and diabetic foot severity.

**Methods:**

A total of 367 diabetic foot patients were retrospectively enrolled, and divided into mild diabetic foot group (Wagner grade 0-2)(n=239) and severe diabetic foot group (Wagner grade 3-5)(n=128). Clinical baseline data and laboratory examination data of the patients were collected, and the HALP score, mSII and mSIRI were calculated. The differences in HALP score, mSII and mSIRI between patients with mild and severe diabetic foot were compared. Logistic regression analysis was performed to explore the associations of HALP score, mSII and mSIRI with severe diabetic foot.

**Results:**

The severe diabetic foot group had significantly higher levels of mSII (*p* < 0.001) and mSIRI (*p* < 0.001), while the HALP level was notably lower (*p* < 0.001) than mild diabetic foot group. The cutoff value of mSII was 5482.235, while mSIRI had a cutoff of 1465.115 and HALP had a cutoff of 16.8 by ROC analysis. Logistic regression analysis demonstrated that reduced HALP [≤16.8 vs. >16.8; odds ratio (OR)=2.113, 95% confidence interval (CI)=1.093-4.086, *p* = 0.026] were associated with severe diabetic foot after adjusting for other confounding factors (age, gender, history of alcohol abuse, history of smoking, history of cerebral infarction, history of coronary artery disease, and history of hypertension), but mSII (*p* = 0.227) and mSIRI (*p* = 0.771) not.

**Conclusions:**

The HALP score correlates with diabetic foot severity and can be used as a potential reference indicator for evaluating the severity of diabetic foot; mSII and mSIRI are not associated with the severity of diabetic foot.

## 1 Introduction

Diabetic foot represents one of the most debilitating chronic complications of diabetes mellitus (1, 2). Its core pathological foundations include peripheral neuropathy, peripheral arterial disease, and infection. Clinically, it predominantly manifests as foot ulcers and gangrene, with severe cases potentially progressing to lower extremity amputation, thereby placing a substantial burden on patients’ families and the public healthcare system (3, 4). Epidemiological statistics indicate that the global incidence of diabetic foot ulcer (DFU) among diabetic patients ranges from 15% to 25% (5). Furthermore, over 85% of non-traumatic amputations are associated with diabetic foot (6). Characterized by high morbidity, disability, and recurrence rates, diabetic foot has emerged as a major clinical challenge in the management of diabetes (7). The prognosis of diabetic foot is closely correlated with the severity of the condition (8). Early accurate assessment of the severity and timely intervention are the key factors for reducing the amputation rate and improving the prognosis. Therefore, the identification of convenient biomarkers that can accurately evaluate diabetic foot severity and guide clinical intervention is of great clinical significance for refining diagnostic and therapeutic strategies and improving long-term patient outcomes.

In recent years, the regulatory role of systemic inflammatory response (9) and nutritional metabolic status (10) on the onset and progression of diabetic foot have garnered extensive research attention. The pathological process of diabetic foot is not only associated with local tissue damage and repair disorders, but also closely linked to systemic immune-inflammatory imbalance and insufficient nutritional reserves (9, 10). Chronic hyperglycemia-triggered oxidative stress and inflammatory responses aggravate vascular endothelial damage and neurological degeneration (11, 12), while nutritional deficiencies further compromise tissue repair capacity and immune defense function, which together accelerate disease progression (13). Based on this, a series of composite scoring systems integrating inflammatory indicators and nutritional parameters have been gradually applied to the assessment and prognostic prediction of diabetic foot, providing an important reference for clinical diagnosis and treatment (14, 15).

As a comprehensive index integrating nutritional status and immune function, the Hemoglobin-Albumin-Lymphocyte-Platelet (HALP) score consists of core parameters that are closely associated with systemic health status. Specifically, hemoglobin reflects systemic oxygen-carrying capacity and anemia severity; albumin acts as a pivotal biomarker for assessing nutritional reserves; lymphocytes participate in immune response regulation; and platelets are tightly linked to coagulation function and inflammatory responses (16, 17). Accumulated studies have verified that the HALP score is significantly correlated with diabetic foot ulcers (15, 18). Patients with a low HALP score have a poorer prognosis, suggesting that this score may hold potential value in the assessment of diabetic foot severity. Meanwhile, systemic immune-inflammation-related indices have become a prevailing research hotspot in disease assessment. The modified Systemic Immune-Inflammatory Index (mSII) and modified Systemic Inflammatory Response Index (mSIRI) integrate the counts of inflammation-associated cells, including neutrophils, lymphocytes, and platelets, to quantify the intensity of systemic inflammatory responses. Both indices have shown reliable application value in disease grading and prognostic prediction for a variety of clinical disorders (19).

Existing studies have preliminarily revealed the importance of inflammation- and nutrition-related composite indicators in the diagnosis and treatment of diabetic foot (14, 15), yet the evaluative value of different indicators varies. With respect to the role of the HALP score, mSII, and mSIRI in assessing the severity of diabetic foot, clinical evidence remains scarce to date, and whether these indicators can effectively reflect disease severity remains unclear. Clarifying the correlation between the HALP score, mSII, mSIRI and diabetic foot severity can provide evidence for the clinical construction of a more accurate disease assessment system. In view of this, the present study focuses on the aforementioned three indicators, aiming to explore the correlation between the HALP score, mSII, mSIRI and diabetic foot severity, clarify the application value of different indicators in diabetic foot assessment, and provide theoretical support for optimizing clinical diagnosis and treatment strategies.

## 2 Materials and methods

### 2.1 Subjects

Patients with diabetic foot who were treated at Affiliated Guangdong Hospital of Integrated Traditional Chinese and Western Medicine of Guangzhou University of Chinese Medicine from April 2015 to October 2023 were retrospectively enrolled as the research subjects. Inclusion criteria: (1) conforming to the diagnostic criteria for diabetes mellitus specified in the Guidelines for the Prevention and Treatment of Diabetes Mellitus in China; (2) the diagnosis and grading of diabetic foot meeting the Wagner Grading System (Grades 0-5); and (3) complete clinical data, including general demographic information, laboratory test results, and medical records related to foot lesions. Exclusion criteria: (1) complicated with other severe infectious diseases (such as sepsis, pneumonia); (2) complicated with malignant tumors, hematological diseases, or autoimmune diseases; (3) complicated with severe hepatic or renal insufficiency; (4) having received immunosuppressants or glucocorticoid therapy within the past month; and (5) incomplete clinical data.

This study was approved by the Medical Ethics Committee of Affiliated Guangdong Hospital of Integrated Traditional Chinese and Western Medicine of Guangzhou University of Chinese Medicine, and all patients or their family members signed the informed consent forms. All patients in this study were under regular diabetes follow-up. Follow-up was performed by the diabetes care team, with regular monitoring of blood glucose, HbA1c, and other relevant indicators, and timely adjustment of treatment strategies based on the follow-up results. The follow-up process was standardized and documented in detail.

### 2.2 Data collection and classification of indicators

General clinical data of the patients were extracted from the hospital electronic medical record system, including gender, age, history of alcohol abuse, history of smoking, History of cerebral infarction, history of coronary artery disease, history of hypertension, duration of diabetes mellitus, and the status of diabetic complications (diabetic nephropathy, diabetic retinopathy, and diabetic peripheral neuropathy). Meanwhile, the results of the Wagner grading for the patients’ diabetic foot were recorded.

Individuals with a hypertension history refer to those who have previously received a definite diagnosis of hypertension or are currently taking oral antihypertensive medications. Hypertension is identified if the average systolic blood pressure (SBP) is higher than 140 mmHg and/or the average diastolic blood pressure (DBP) exceeds 90 mmHg (20). Diagnostic criteria for diabetes mellitus are as follows: presence of diabetic symptoms accompanied by a random plasma glucose level ≥11.1 mmol/L, a fasting plasma glucose (FPG) level ≥7.0 mmol/L, or a 2-hour plasma glucose level ≥11.1 mmol/L after a 75-g oral glucose tolerance test (OGTT) (21). Patients with a smoking history are defined as those who have smoked no fewer than one cigarette per day for at least one year, or those who have quit smoking for less than six months. Those with an alcohol consumption history refer to individuals who drink alcohol at least once a week.

In this study, patients with Wagner grade 0–2 diabetic foot were assigned to the mild diabetic foot group, while those with Wagner grade 3–5 were categorized into the severe diabetic foot group.

### 2.3 Detection of laboratory indicators and calculation of the HALP score, mSII, and mSIRI

All patients were required to fast overnight and have 5 mL of cubital venous blood drawn early in the morning on the second day after admission. The blood samples were placed in EDTA anticoagulant tubes and standard serum separation tubes, followed by the completion of relevant indicator tests. Peripheral venous blood samples were collected from all participants in the early morning after an overnight fast. Albumin, hemoglobin, lymphocyte count, platelet count, and neutrophil count were determined using an automatic biochemical analyzer (Beckman Coulter AU5800, Beckman Coulter Inc., USA) and an automatic blood cell analyzer (Sysmex XE-2100 hematology analyzer, Sysmex Corporation, Japan) according to the manufacturer’s standard protocols. All laboratory examinations were performed by experienced technicians who were blinded to the clinical data of the study subjects to ensure the accuracy and reliability of the test results.

Three composite indicators, namely mSII, mSIRI, and HALP, were computed using the equations outlined below:

mSII=platelet×neutrophil/lymphocyte×ln(albumin) (19, 22);

mSIRI=platelet×neutrophil/lymphocyte (19, 22);

The HALP score was derived from hemoglobin (g/L), serum albumin (g/L), lymphocyte count (×10^9^/L) and platelet count (×10^9^/L), with the following equation: HALP = hemoglobin × albumin × lymphocyte count/platelet count (23, 24).

### 2.4 Statistical analysis

Continuous variables that conformed to a normal distribution were expressed as mean ± standard deviation, whereas those that did not meet the normal distribution requirement were described as median (25th percentile, 75th percentile). Count data were presented as case count (percentage). Receiver operating characteristic (ROC) curve analysis was utilized to determine the optimal cutoff values of mSII, mSIRI, and HALP for distinguishing severe diabetic foot cases stratified by the Wagner grade. The Chi-square test was adopted to compare the clinical characteristics between patients with mild and severe diabetic foot. Nonparametric tests were used to assess the association between severe diabetic foot and the three indices (mSII, mSIRI, HALP). Logistic regression analysis was employed to investigate the correlations between these indicators and severe diabetic foot. A two-tailed *p* value less than 0.05 was regarded as statistically significant. All data analyses were conducted with SPSS statistical software (Version 26.0, IBM Inc., United States of America).

## 3 Results

### 3.1 Clinicopathological features of patients with diabetes mellitus

A total of 367 patients were enrolled in this study, including 93 (25.3%) were aged ≤60 years and 274 (74.7%) were aged >60 years. The cohort consisted of 205 (55.9%) male patients and 162 (44.1%) female patients. In terms of clinical comorbidities and lifestyle history, the proportions of patients with history of alcohol abuse, history of smoking, history of cerebral infarction, history of coronary artery disease, and history of hypertension were 7.6% (28/367), 16.1% (59/367), 15.3% (56/367), 16.6% (61/367), and 63.8% (234/367), respectively. Regarding the duration of diabetes mellitus, 108 patients had a disease course of <5 years, 193 had a course of 5–15 years, and 54 had a course of >15 years. A total of 111 (30.2%), 44 (12.0%), and 162 (44.1%) patients were complicated with diabetic nephropathy, diabetic retinopathy, and diabetic peripheral neuropathy, respectively. Based on the Wagner grade, patients at grade 2 accounted for the largest proportion (36.2%, 133/367), followed by those at grade 1 (27.5%, 101/367), grade 4 (17.2%, 63/367), grade 3 (16.6%, 61/367), grade 0 (1.4%, 5/367), and grade 5 (1.1%, 4/367) (**Table 1**).

**Table 1:** Clinicopathological features of patients with diabetic foot.

Clinicopathological features	Patients with diabetic foot (n=367)
Gender	
Male, n (%)	205 (55.9%)
Female, n (%)	162 (44.1%)
Age (Years)	
≤60, n (%)	93 (25.3%)
>60, n (%)	274 (74.7%)
History of alcohol abuse	
No, n (%)	339 (92.4%)
Yes, n (%)	28 (7.6%)
History of smoking	
No, n (%)	308 (83.9%)
Yes, n (%)	59 (16.1%)
History of cerebral infarction	
No, n (%)	311 (84.7%)
Yes, n (%)	56 (15.3%)
History of coronary artery disease	
No, n (%)	306 (83.4%)
Yes, n (%)	61 (16.6%)
History of hypertension	
No, n (%)	133 (36.2%)
Yes, n (%)	234 (63.8%)
Duration of diabetes mellitus	
<5 years, n (%)	108 (29.4%)
5–15 years, n (%)	193 (52.6%)
>15 years, n (%)	54 (14.7%)
Unknown, n (%)	12 (3.3%)
Complicated with diabetic nephropathy	
No, n (%)	256 (69.8%)
Yes, n (%)	111 (30.2%)
Complicated with diabetic retinopathy	
No, n (%)	323 (88.0%)
Yes, n (%)	44 (12.0%)
Complicated with diabetic peripheral neuropathy	
No, n (%)	205 (55.9%)
Yes, n (%)	162 (44.1%)
Wagner grade	
Grade 0, n (%)	5 (1.4%)
Grade 1, n (%)	101 (27.5%)
Grade 2, n (%)	133 (36.2%)
Grade 3, n (%)	61 (16.6%)
Grade 4, n (%)	63 (17.2%)
Grade 5, n (%)	4 (1.1%)

### 3.2 Comparison of clinical characteristics between patients with mild and severe diabetic foot stratified by Wagner grade

**Figure 1:**
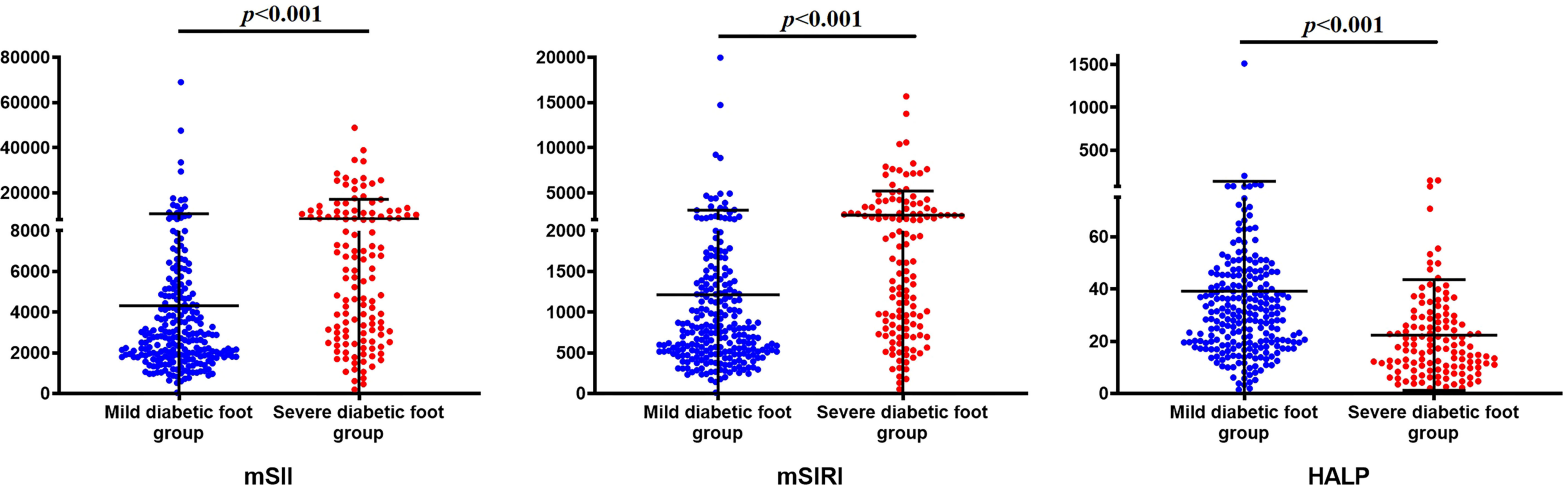
The levels of mSII, mSIRI, and HALP score in patients with mild and severe diabetic foot stratified by Wagner grade.

In this study, 239 patients were diagnosed with mild diabetic foot, while 128 cases were classified as severe diabetic foot. A statistically significant difference was observed in the prevalence of diabetic peripheral neuropathy between the two groups; specifically, the proportion of patients complicated with diabetic peripheral neuropathy was notably higher in the mild diabetic foot group than in the severe diabetic foot group (50.6% vs. 32.0%, χ^2^ = 11.691, *p* = 0.001). No statistically significant differences were observed between the two groups in terms of gender, age, history of alcohol abuse, history of smoking, history of cerebral infarction, history of coronary artery disease, history of hypertension, duration of diabetes mellitus, complicated with diabetic nephropathy, or complicated with diabetic retinopathy (all *p*>0.05) (**Table 2**). Compared with the mild diabetic foot group, the severe diabetic foot group had significantly higher levels of mSII (*p* < 0.001) and mSIRI (*p* < 0.001), while the HALP level was notably lower (*p* < 0.001) (**Figure 1**).

**Table 2:** Comparison of clinical characteristics between patients with mild and severe diabetic foot stratified by Wagner grade.

Clinicopathological features	Patients with mild diabetic foot (n=239)	Patients with severe diabetic foot (n=128)	*p* (χ^2^)
Gender			
Male, n (%)	126 (52.7%)	79 (61.7%)	0.100 (χ^2^ = 2.738)
Female, n (%)	113 (47.3%)	49 (38.3%)	
Age (Years)			
≤60, n (%)	60 (25.1%)	33 (25.8%)	0.900 (χ^2^ = 0.020)
>60, n (%)	179 (74.9%)	95 (74.2%)	
History of alcohol abuse			
No, n (%)	217 (90.8%)	122 (95.3%)	0.150 (χ^2^ = 2.414)
Yes, n (%)	22 (9.2%)	6 (4.7%)	
History of smoking			
No, n (%)	199 (83.3%)	109 (85.2%)	0.659 (χ^2^ = 0.221)
Yes, n (%)	40 (16.7%)	19 (14.8%)	
History of cerebral infarction			
No, n (%)	205 (85.8%)	106 (82.8%)	0.543 (χ^2^ = 0.565)
Yes, n (%)	34 (14.2%)	22 (17.2%)	
History of coronary artery disease			
No, n (%)	205 (85.8%)	101 (78.9%)	0.106 (χ^2^ = 2.837)
Yes, n (%)	34 (14.2%)	27 (21.1%)	
History of hypertension			
No, n (%)	92 (38.5%)	41 (32.0%)	0.255 (χ^2^ = 1.507)
Yes, n (%)	147 (61.5%)	87 (68.0%)	
Duration of diabetes mellitus			
<5 years, n (%)	69 (28.9%)	39 (30.5%)	0.194 (χ^2^ = 3.345)
5–15 years, n (%)	121 (50.6%)	72 (56.3%)	
>15 years, n (%)	41 (17.2%)	13 (10.2%)	
Complicated with diabetic nephropathy			
No, n (%)	171 (71.5%)	85 (66.4%)	0.341 (χ^2^ = 1.045)
Yes, n (%)	68 (28.5%)	43 (33.6%)	
Complicated with diabetic retinopathy			
No, n (%)	211 (88.3%)	112 (87.5%)	0.867 (χ^2^ = 0.049)
Yes, n (%)	28 (11.7%)	16 (12.5%)	
Complicated with diabetic peripheral neuropathy			
No, n (%)	118 (49.4%)	87 (68.0%)	0.001 (χ^2^ = 11.691)
Yes, n (%)	121 (50.6%)	41 (32.0%)	

### 3.3 Univariate logistic regression analysis of associated factors with severe diabetic foot

**Figure 2:**
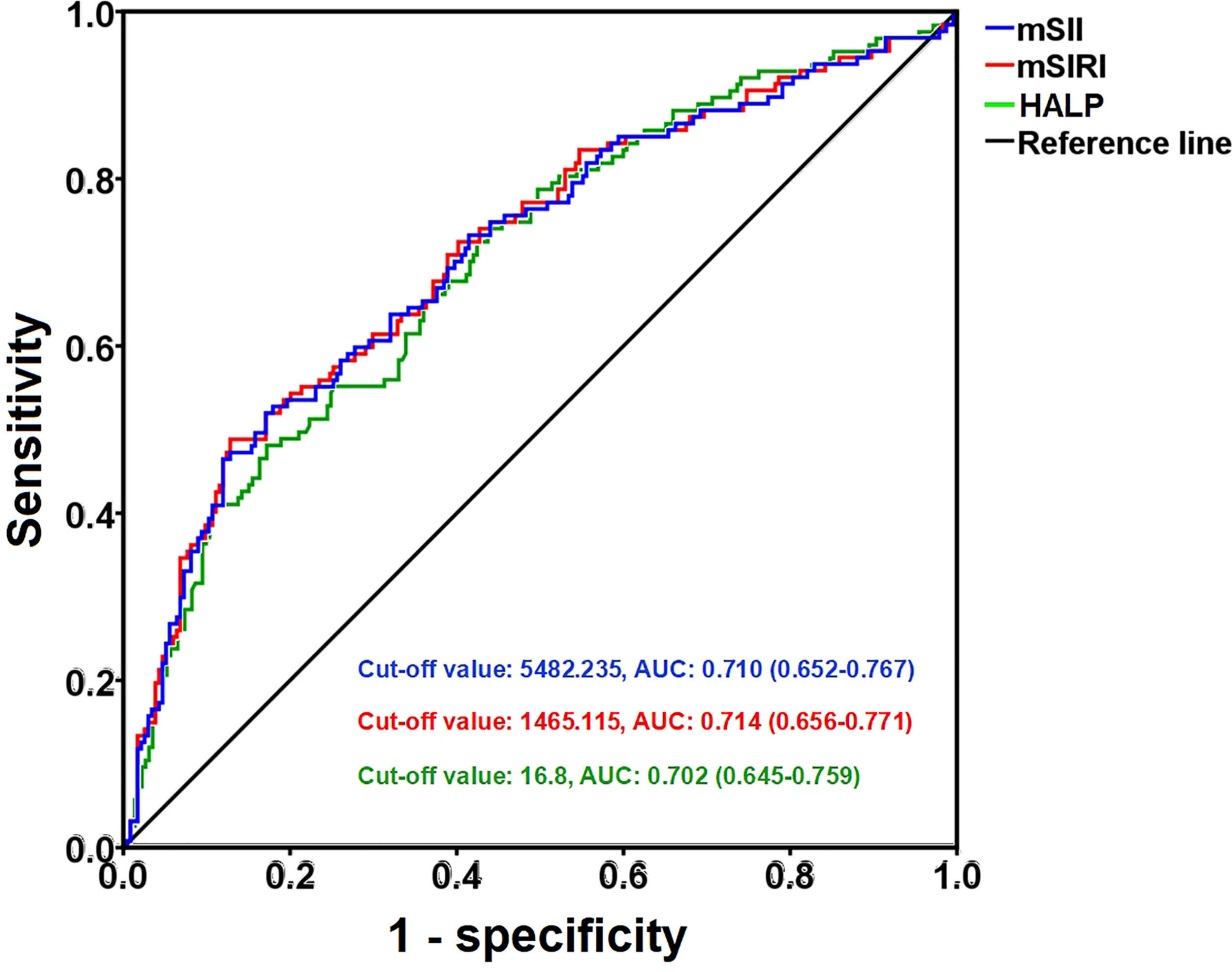
The ROC curve of mSII, mSIRI, and HALP score based on the severe diabetic foot.

ROC curve analysis was performed with severe diabetic foot as the endpoint to determine the critical values of mSII, mSIRI, and HALP. The cutoff value of mSII was 5482.235 [sensitivity=52.8%, specificity=82.1%, area under the ROC curve (AUC)=0.710], while mSIRI had a cutoff of 1465.115 (sensitivity=53.1%, specificity=80.8%, AUC = 0.714) and HALP had a cutoff of 16.8 (sensitivity=48.0%, specificity=82.5%, AUC = 0.702) (**Figure 2**).

Univariate logistic regression analysis revealed that elevated mSII [>5482.235 vs. ≤5482.235; odds ratio (OR)=5.105, 95% confidence interval (CI)=3.151-8.271, *p* < 0.001], increased mSIRI (>1465.115 vs. ≤1465.115; OR = 4.755, 95% CI = 2.962-7.633, *p* < 0.001), and reduced HALP (≤16.8 vs. >16.8; OR = 4.351, 95% CI = 2.680-7.063, *p* < 0.001) were significantly associated with severe diabetic foot (**Table 3**).

**Table 3:** Univariate logistic regression analysis of associated factors with severe diabetic foot.

Variables	OR	95% CI	*p* values
Gender (male/female)	1.446	0.933-2.240	0.099
Age (>60/≤60, years old)	0.965	0.590-1.579	0.887
History of alcohol abuse (yes/no)	0.485	0.191-1.229	0.127
History of smoking (yes/no)	0.867	0.479-1.571	0.638
History of cerebral infarction (yes/no)	1.251	0.697-2.247	0.453
History of coronary artery disease (yes/no)	1.612	0.922-2.818	0.094
History of hypertension (yes/no)	1.328	0.844-2.090	0.220
Duration of diabetes mellitus			
<5 years, n (%)	1.000 (reference)	–	–
5–15 years, n (%)	1.053	0.645-1.717	0.837
>15 years, n (%)	0.561	0.268-1.172	0.124
Complicated with diabetic nephropathy (yes/no)	1.272	0.801-2.019	0.307
Complicated with diabetic retinopathy (yes/no)	1.077	0.559-2.074	0.826
Complicated with diabetic peripheral neuropathy (yes/no)	0.460	0.293-0.720	0.001
mSII (>5482.235/≤5482.235)	5.105	3.151-8.271	<0.001
mSIRI (>1465.115/≤1465.115)	4.755	2.962-7.633	<0.001
HALP (≤16.8/>16.8)	4.351	2.680-7.063	<0.001

OR, odds ratio; CI, confidence interval.

### 3.4 Multivariate logistic regression analysis of associated factors with severe diabetic foot

Multivariate logistic regression analysis demonstrated that reduced HALP (≤16.8 vs. >16.8; OR = 2.113, 95% CI = 1.093-4.086, *p* = 0.026) remained associated with severe diabetic foot after adjusting for: age, gender, history of alcohol abuse, history of smoking, history of cerebral infarction, history of coronary artery disease, and history of hypertension, but mSII (*p* = 0.227) and mSIRI (*p* = 0.771) were no longer associated with severe diabetic foot (**Table 4**).

**Table 4:** Multivariate logistic regression analysis of associated factors with severe diabetic foot.

Variables	Adjusted OR	95% CI	*p* values
Duration of diabetes mellitus			
<5 years, n (%)	1.000 (reference)	–	–
5–15 years, n (%)	1.001	0.559-1.792	0.997
>15 years, n (%)	0.597	0.254-1.402	0.236
Complicated with diabetic nephropathy (yes/no)	1.034	0.581-1.839	0.910
Complicated with diabetic retinopathy (yes/no)	1.228	0.533-2.829	0.630
Complicated with diabetic peripheral neuropathy (yes/no)	0.496	0.295-0.834	0.008
mSII (>5482.235/≤5482.235)	4.306	0.403-46.001	0.227
mSIRI (>1465.115/≤1465.115)	0.707	0.068-7.334	0.771
HALP (≤16.8/>16.8)	2.113	1.093-4.086	0.026

OR, odds ratio; CI, confidence interval.

Adjust for: age, gender, history of alcohol abuse, history of smoking, history of cerebral infarction, history of coronary artery disease, and history of hypertension.

## 4 Discussion

This study investigated the associations between the HALP score, mSII, mSIRI, and the severity of diabetic foot. The results indicated that HALP score was significantly correlated with diabetic foot severity, whereas mSII and mSIRI showed no obvious association with it. These findings highlight the relationship of composite systemic inflammatory−nutritional related indices and diabetic foot severity, and may provide supportive evidence for risk stratification and clinical monitoring of diabetic foot.

The significant correlation between the HALP score and diabetic foot severity is consistent with the core regulatory mechanism of the “nutrition-immunity-tissue repair” axis underlying diabetic foot pathogenesis (25–27). The HALP score integrates four indicators: hemoglobin, albumin, lymphocytes, and platelets. Among these, hemoglobin and albumin serve as key biomarkers reflecting the body’s nutritional reserves; lymphocytes represent the level of cellular immune function; and platelets are involved in inflammatory responses and tissue healing processes. As a severe chronic complication of diabetes mellitus, diabetic foot is closely related to systemic nutritional deterioration (such as albumin, prealbumin, total protein, hemoglobin, and micronutrients), immune imbalance, and microcirculation disturbance caused by long−term hyperglycemia (28). In patients with severe diabetic foot, particularly those complicated with ulcers, infections or gangrene, the body maintains a state of persistent hypercatabolism and hyperinflammation (29). On the one hand, this status enhances protein catabolism and reduces albumin synthesis (30, 31); on the other hand, it impairs the hematopoietic microenvironment, leading to decreased hemoglobin levels and consequent nutritional deficiency (32). In addition, a potential interplay exists between immune dysfunction and microthrombosis in diabetic foot. Chronic hyperglycemia induces persistent low−grade inflammation and immune imbalance, which impair vascular endothelial function, trigger platelet activation and aggregation, and promote the formation of microthrombi (33). Occlusion of microvessels by microthrombi exacerbates tissue ischemia and hypoxia, further weakening local immune defense and delaying tissue repair (34). This reciprocal interaction between immune disturbance and microthrombosis may jointly participate in the occurrence and deterioration of diabetic foot, which warrants further verification in mechanistic studies.

On the other hand, persistent inflammatory responses can inhibit lymphocyte proliferation and activity, leading to cellular immune deficiency (35). This impairment hinders the effective clearance of invasive pathogens, further exacerbating local infection and tissue damage in diabetic foot lesions (36). Meanwhile, as crucial participants in inflammatory responses, platelets exhibit quantitative changes that may be associated with the formation of local microthrombi, tissue hypoxia, and necrosis (37). Therefore, by comprehensively reflecting the body’s nutritional status, immune function, and inflammatory levels, the HALP score can accurately capture the core pathophysiological changes during the progression of diabetic foot, thus exhibiting a significant correlation with disease severity. These findings are consistent with the value of HALP score as a prognostic indicator for various chronic diseases reported in previous studies, further confirming its universality in the assessment of chronic consumptive diseases and providing a convenient and comprehensive composite indicator for diabetic foot evaluation (38–40). Compared with single biomarkers, the HALP score integrates multidimensional pathophysiological information, enables a more holistic evaluation of the patient’s systemic status, and exhibits superior efficacy in stratifying the severity of diabetic foot.

Furthermore, this study found that neither the mSII nor the mSIRI exhibited any correlation with the severity of diabetic foot. This result was inconsistent with our expectations, which may be attributed to the combined effects of multiple factors, including the inherent characteristics of the indicators themselves, the pathological features of diabetic foot, and the demographic traits of the study population. Calculated primarily based on inflammation-related cell counts, mSII and mSIRI are dedicated to reflecting the intensity of systemic inflammatory responses and characterizing the balance between pro-inflammatory and immune cell populations. Notably, inflammation in diabetic foot follows a distinct pattern, featured by dominant local lesions and secondary systemic inflammatory activation (41). In the early stage, diabetic foot is centered on local microcirculation disturbance and neuropathy, with relatively mild systemic inflammatory responses. When diabetic foot progresses to the severe stage, the elevation of systemic inflammatory levels may also be affected by factors such as the control status of local infection and individual differences in inflammatory responses, which weakens the correlation between systemic inflammatory indicators (mSII, mSIRI) and the severity of local lesions (42). Furthermore, patients with diabetic foot often have comorbidities such as hypertension, nephropathy, and cardiovascular diseases (43, 44). These underlying conditions can themselves induce abnormal levels of mSII and mSIRI, thereby interfering with the correlation between these indices and diabetic foot severity. Meanwhile, in contrast to the HALP score, mSII and mSIRI do not incorporate nutrition-related indicators. Notably, mSII and mSIRI are purely inflammatory−based indices derived from leukocyte and platelet counts, and do not include any nutritional components. By contrast, the HALP score incorporates hemoglobin and albumin, two key biomarkers of systemic nutritional status. Albumin represents protein synthesis and nutritional reserve, while hemoglobin reflects anemia and nutritional deficiency. By integrating nutritional and immune− inflammatory information, HALP may provide a more comprehensive evaluation of the overall physiological status in patients with diabetic foot, which may account for its stronger association with disease severity. Given that nutritional status is a key factor influencing the progression and wound healing of diabetic foot, this may prevent the two indices from fully capturing the core pathophysiological processes underlying diabetic foot progression, thus reducing their value in assessing disease severity.

It should be noted that in previous studies, the mSII and mSIRI have demonstrated certain value in the assessment of inflammation-related diseases (19). However, this study failed to yield similar conclusions, which might be attributed to the specificity of the study population. Inconsistent with previous studies, mSII and mSIRI showed no independent predictive value in our multivariate analysis. This discrepancy may be partly explained by racial and genetic differences between study populations. Ethnic variations in genetic background, inflammatory response patterns, and metabolic characteristics among different populations in various studies may influence the associations between these inflammatory indices and diseases, leading to the inconsistent results observed in the present study. The diabetic foot patients enrolled in this study were likely dominated by local lesions, with systemic inflammatory responses failing to reach a state of significant activation. Consequently, the magnitude of changes in mSII and mSIRI was relatively small, making it impossible to effectively distinguish between diabetic foot patients with different severity levels.

In the present study, the proportion of diabetic peripheral neuropathy was significantly higher in the mild diabetic foot group than in the severe group (50.6% vs. 32.0%, χ^2^ = 11.691, *p* = 0.001). Although this result seems counterintuitive, it may be explained by the fact that severe diabetic foot is often dominated by severe ischemia, infection, and tissue necrosis, which may mask the clinical manifestations of peripheral neuropathy and lead to its underestimation. In addition, with the deterioration of diabetic foot, severe vascular lesions may gradually replace neuropathy as the main driving factor for disease progression. Therefore, this finding reflects the complex interaction between neuropathy, vascular disease and infection in diabetic foot, rather than contradicting the important role of neuropathy in its occurrence and development. These findings warrant further validation in larger prospective cohorts.

From a clinical perspective, the findings of this study clarify the advantages of the HALP score in the assessment of diabetic foot, thereby providing a practical evaluation tool for clinical practice. Clinicians can conveniently determine the disease severity and overall physical status of diabetic foot patients based on laboratory indicators. For diabetic foot patients with low HALP scores, this indicates insufficient nutritional reserves and impaired immune function. Clinically, priority should be given to strengthening nutritional support therapy, regulating immune function, actively controlling local infections, and improving microcirculation, so as to delay disease progression and promote ulcer healing. However, this study has certain limitations. Firstly, as a single-center, cross-sectional study, it cannot clarify the causal relationship between the HALP score and the progression of diabetic foot, nor can it observe the predictive value of the index for prognosis. Secondly, the influence of baseline medication use on index levels was not excluded. Thirdly, the relatively limited sample size may compromise the extrapolability of the results. Future multicenter prospective cohort studies with larger sample sizes are therefore warranted to dynamically monitor changes in the HALP score, mSII, and mSIRI and clarify their predictive values for key diabetic foot prognostic outcomes, including ulcer healing rate, amputation rate, and mortality. Additionally, in-depth exploration of the molecular mechanisms underlying the effect of the HALP score on diabetic foot progression is warranted, which may provide a theoretical basis for targeted therapy.

## 5 Conclusions

The HALP score can effectively reflect the severity of diabetic foot and serves as an optimal composite indicator for diabetic foot assessment. In contrast, mSII and mSIRI show no significant correlation with diabetic foot severity, indicating their limited application value in this condition. The findings of this study provide valuable reference information for the assessment of diabetic foot, guidance in the formulation of treatment regimens, and prediction of disease prognosis.

## Data Availability

The original contributions presented in the study are included in the article/supplementary material. Further inquiries can be directed to the corresponding author.

## References

[1] Flekač M . Diabetic foot attack. Cas Lek Cesk. (2024) 163:194–6 39516021

[2] Pérez-Panero AJ Ruiz-Muñoz M . Diabetic foot disease: a systematic literature review of patient-reported outcome measures. Qual Life Res. (2021) 30:3395–405. doi: 10.1007/s11136-021-02892-4. PMID: 34109501

[3] Bandyk DF . The diabetic foot: pathophysiology, evaluation, and treatment. Semin Vasc Surg. (2018) 31:43–8. doi: 10.1053/j.semvascsurg.2019.02.001. PMID: 30876640

[4] Edmonds M Manu C Vas P . The current burden of diabetic foot disease. J Clin Orthop Trauma. (2021) 17:88–93. doi: 10.1016/j.jcot.2021.01.017. PMID: 33680841 PMC7919962

[5] Rehman ZU Khan J Noordin S . Diabetic foot ulcers: contemporary assessment and management. J Pak Med Assoc. (2023) 73:1480–7. doi: 10.47391/JPMA.6634. PMID: 37469062

[6] Lee JH Yoon JS Lee HW Won KC Moon JS Chung SM . Risk factors affecting amputation in diabetic foot. Yeungnam Univ J Med. (2020) 37:314–20. doi: 10.12701/yujm.2020.00129. PMID: 32370489 PMC7606965

[7] Castellino LM Crisologo PA Chhabra A Öz OK . Diabetic foot infections. Infect Dis Clin North Am. (2025) 39:465–82. doi: 10.1016/j.idc.2025.02.014. PMID: 40204567

[8] Yu J Kim JH Kim B Han K Lee SH Kim MK . The severity of diabetes and the risk of diabetic foot amputation: a national cohort study. Endocrinol Metab (Seoul). (2025) 40:574–82. doi: 10.3803/EnM.2024.2266. PMID: 40229968 PMC12409162

[9] Hu Y Xiong F Zhao L Wan F Hu X Shen Y . Association between systemic inflammatory response index and diabetic foot ulcer in the US population with diabetes in the NHANES: a retrospective cross-sectional study. Int J Low Extrem Wounds. (2025) 24:611–20. doi: 10.1177/15347346251324478. PMID: 40080867

[10] Li J Shi H Cao Y . Identification of disulfidptosis-related genes in immunity and immunotherapy in diabetic foot ulcer. Ann Med Surg. (2026) 88:1402–14. doi: 10.1097/MS9.0000000000003859. PMID: 41675755 PMC12889416

[11] Vujčić S Kotur-Stevuljević J Vekić J Perović-Blagojević I . Oxidative stress and inflammatory biomarkers in patients with diabetic foot. Med (Kaunas). (2022) 58:1866. doi: 10.3390/medicina58121866. PMID: 36557068 PMC9785583

[12] Sangeeta S Siripuram C Konka S Vaithilingam K Periasamy P Velu RK . Biomarkers of inflammation, oxidative stress, and endothelial dysfunction in early detection of diabetic foot ulcers. Cureus. (2025) 17:e82174. doi: 10.7759/cureus.82174. PMID: 40364880 PMC12072064

[13] Batarbekova S Zhunussova D Derbissalina G Bekbergenova Z Maksimova N Umbetzhanova A . Micronutrient status of patients with diabetic foot: a systematic review. Asia Pac J Clin Nutr. (2025) 34:487–501. doi: 10.6133/apjcn.202508_34(4).0001. PMID: 40738717 PMC12310431

[14] Liu B Wang L He Y . Association between systemic immune-inflammatory index (SIRI) and diabetic foot ulcers in individuals with diabetes: evidence from the NHANES. Int J Low Extrem Wounds. (2024), 15347346241309180. doi: 10.1177/15347346241309180. PMID: 39699113

[15] Li Z Guo H Fu Z Li D Zhang Y Zhu R . Association between hemoglobin, albumin, lymphocyte, and platelet score and diabetic foot ulcer: a cross-sectional study. Int J Low Extrem Wounds. (2025), 15347346251355578. doi: 10.1177/15347346251355578. PMID: 40605505

[16] Li H Zhou Y Zhang X Yao R Li N . The relationship between hemoglobin, albumin, lymphocyte, and platelet (HALP) score and 28-day mortality in patients with sepsis: a retrospective analysis of the MIMIC-IV database. BMC Infect Dis. (2025) 25:333. doi: 10.1186/s12879-025-10739-3. PMID: 40065235 PMC11892195

[17] He Y Ma Z Chen X Wang J Chen X Deng Z . Association between hemoglobin, albumin, lymphocyte, and platelet score and all-cause and cardiovascular mortality among population with diabetes: evidence from the NHANES 2003-2016. Diabetes Res Clin Pract. (2025) 224:112212. doi: 10.1016/j.diabres.2025.112212. PMID: 40345595

[18] Chen H Zhou Y Dai J . Association of inflammation and nutrition-based indicators and diabetic foot ulcers: a cross-sectional study and a retrospective study. Front Endocrinol (Lausanne). (2025) 16:1654831. doi: 10.3389/fendo.2025.1654831. PMID: 41036136 PMC12479306

[19] Cai J Rao H Li X Luo J Wang Z Liu D . Predictive value of the modified comprehensive immunoinflammatory indices for hemorrhagic transformation in ischemic stroke patients undergoing thrombolysis: a retrospective study. Int J Gen Med. (2025) 18:6353–63. doi: 10.2147/IJGM.S545665. PMID: 41141888 PMC12553340

[20] Wang Z Chen Z Zhang L Wang X Hao G Zhang Z . Status of hypertension in China: results from the China Hypertension Survey, 2012-2015. Circulation. (2018) 137:2344–56. doi: 10.1161/CIRCULATIONAHA.117.032380. PMID: 29449338

[21] Benhalima K Van Crombrugge P Moyson C Verhaeghe J Vandeginste S Verlaenen H . Risk factor screening for gestational diabetes mellitus based on the 2013 WHO criteria. Eur J Endocrinol. (2019) 180:353–63. doi: 10.1530/EJE-19-0117. PMID: 31120231

[22] Gan Y Zhu R Li J Wang Q Meng X . Modified systemic immune-inflammatory index, modified systemic inflammatory response index and hemoglobin-albumin-lymphocyte-platelet score may serve as markers for evaluating the efficacy of neoadjuvant therapy in breast cancer patients. Front Oncol. (2026) 16:1746164. doi: 10.3389/fonc.2026.1746164. PMID: 41768251 PMC12945815

[23] Jiang T Sun H Xue S Xu T Xia W Wang Y . Prognostic significance of hemoglobin, albumin, lymphocyte, and platelet (HALP) score in breast cancer: a propensity score-matching study. Cancer Cell Int. (2024) 24:230. doi: 10.1186/s12935-024-03419-w. PMID: 38956686 PMC11218366

[24] Azapoğlu Kaymak B Eksioglu M Cimilli Öztürk T Köroğlu M . Hemoglobin, albumin, lymphocyte and platelet score as a novel predictor of mortality and rebleeding in patients with upper gastrointestinal bleeding. Int J Gen Med. (2025) 18:2391–400. doi: 10.2147/IJGM.S520925. PMID: 40352470 PMC12063693

[25] Zhu Y Xu H Wang Y Feng X Liang X Xu L . Risk factor analysis for diabetic foot ulcer-related amputation including Controlling Nutritional Status score and neutrophil-to-lymphocyte ratio. Int Wound J. (2023) 20:4050–60. doi: 10.1111/iwj.14296. PMID: 37403337 PMC10681407

[26] Aragón-Sánchez J Víquez-Molina G López-Valverde ME . Systemic immune-inflammation index in diabetic foot infections and osteomyelitis. Int J Low Extrem Wounds. (2025) 24:1278–80. doi: 10.1177/15347346231179280. PMID: 37264592

[27] Chhillar A Jaiswal A . Hyaluronic acid-based self-healing hydrogels for diabetic wound healing. Adv Healthc Mater. (2025) 14:e2404255. doi: 10.1002/adhm.202404255. PMID: 39722163

[28] Chen H Zhou Y Dai J . Association between three systemic inflammatory biomarkers and diabetic foot ulcer: a cross-sectional study and a clinical retrospective study. Mediators Inflammation. (2026) 2026:7709529. doi: 10.1155/mi/7709529. PMID: 41728362 PMC12921367

[29] Kim J . The pathophysiology of diabetic foot: a narrative review. J Yeungnam Med Sci. (2023) 40:328–34. doi: 10.12701/jyms.2023.00731. PMID: 37797951 PMC10626291

[30] Omo-Okhuasuyi A Jin YF ElHefnawi M . Multimodal identification of molecular factors linked to severe diabetic foot ulcers using artificial intelligence. Int J Mol Sci. (2024) 25:10686. doi: 10.3390/ijms251910686. PMID: 39409014 PMC11476782

[31] Coye TL Suludere MA Kang GE Crisologo PA Malone M Rogers LC . The infected diabetes-related foot: comparison of erythrocyte sedementation rate/albumin and C-reactive protein/albumin ratios with erythrocyte sedimentation rate and C-reactive protein to differentiate bone and soft tissue infections. Wound Repair Regener. (2023) 31:738–44. doi: 10.1111/wrr.13121. PMID: 37843834

[32] Li Y Ju S Li X Li W Zhou S Wang G . Characterization of the microenvironment of diabetic foot ulcers and potential drug identification based on scRNA-seq. Front Endocrinol. (2022) 13:997880. doi: 10.3389/fendo.2022.997880. PMID: 36686438 PMC9845942

[33] Maria B G Aqeela A Polyxenie S Hao P Lynn C S Robert N M . The role of growth factors in the pathogenesis of diabetic retinopathy. Expert Opin Investig Drugs. (2004) 13:1275–93. doi: 10.1517/13543784.13.10.1275. PMID: 15461557

[34] Monica M Srihari L Ryan P H Natalie S Harold D W Imani M . Utilization of thromboelastography with platelet mapping to predict infection and poor wound healing in postoperative vascular patients. Ann Vasc Surg. (2022) 87:213–24. doi: 10.1016/j.avsg.2022.03.008. PMID: 35339591

[35] Mittal R Lemos JRN Chapagain P Hirani K . Interplay of hypoxia, immune dysregulation, and metabolic stress in pathophysiology of type 1 diabetes. Front Immunol. (2025) 16:1599321. doi: 10.3389/fimmu.2025.1599321. PMID: 40534855 PMC12174378

[36] Wu Y Chen T Wang Y Huang M Wang Y Luo Z . New insight into the virulence and inflammatory response of Staphylococcus aureus strains isolated from diabetic foot ulcers. Front Cell Infect Microbiol. (2023) 13:1234994. doi: 10.3389/fcimb.2023.1234994. PMID: 37577369 PMC10416727

[37] Sheng X Hu L Li T Zou Y Fu HY Xiong GP . Clinical efficacy and mechanism of the combination of autologous platelet-rich gel and recombinant human acidic fibroblast growth factor in the management of refractory diabetic foot. Front Endocrinol. (2024) 15:1374507. doi: 10.3389/fendo.2024.1374507. PMID: 39539934 PMC11557328

[38] Demir Y Yamak BA . HALP score as a prognostic biomarker in tricuspid valve surgery: association with in-hospital and long-term mortality. J Inflammation Res. (2025) 18:10637–49. doi: 10.2147/JIR.S517577. PMID: 40799800 PMC12341553

[39] Han H Hu S Du J . Predictive value of the hemoglobin-albumin-lymphocyte-platelet (HALP) index for ICU mortality in patients with acute exacerbations of chronic obstructive pulmonary disease (AECOPD). Intern Emerg Med. (2023) 18:85–96. doi: 10.1007/s11739-022-03132-4. PMID: 36357607

[40] Akbay MO Ernam D . Hemoglobin-albumin-lymphocyte-platelet index and risk of in-hospital mortality in 793 adult patients hospitalized for acute exacerbations of chronic obstructive pulmonary disease. Med Sci Monit. (2025) 31:e947098. doi: 10.12659/MSM.947098. PMID: 40186341 PMC11980517

[41] Lipsky BA Berendt AR Deery HG Embil JM Joseph WS Karchmer AW . Diagnosis and treatment of diabetic foot infections. Plast Reconstr Surg. (2006) 117:212s–38s. doi: 10.1097/01.prs.0000222737.09322.77. PMID: 16799390

[42] Balasubramanian G Vas P Chockalingam N Naemi R . A synoptic overview of neurovascular interactions in the foot. Front Endocrinol. (2020) 11:308. doi: 10.3389/fendo.2020.00308. PMID: 32528410 PMC7256167

[43] Borderie G Foussard N Larroumet A Blanco L Domenge F Mohammedi K . Albuminuric diabetic kidney disease predicts foot ulcers in type 2 diabetes. J Diabetes Complications. (2023) 37:108403. doi: 10.1016/j.jdiacomp.2023.108403. PMID: 36641879

[44] Rossboth S Lechleitner M Oberaigner W . Risk factors for diabetic foot complications in type 2 diabetes-a systematic review. Endocrinol Diabetes Metab. (2021) 4:e00175. doi: 10.1002/edm2.175. PMID: 33532615 PMC7831214

